# The biguanide polyamine analog verlindamycin promotes differentiation in neuroblastoma via induction of antizyme

**DOI:** 10.1038/s41417-021-00386-6

**Published:** 2021-09-14

**Authors:** Zuzanna Urban-Wójciuk, Amy Graham, Karen Barker, Colin Kwok, Yordan Sbirkov, Louise Howell, James Campbell, Patrick M. Woster, Evon Poon, Kevin Petrie, Louis Chesler

**Affiliations:** 1 Division of Clinical Studies, Institute of Cancer Research, London, UK; 2 Division of Cancer Therapeutics, Institute of Cancer Research, London, UK; 3 School of Natural Sciences, University of Stirling, Stirling, UK; 4 Cell Imaging Facility, Institute of Cancer Research, London, UK; 5 Bioinformatics Core Facility, Institute of Cancer Research, London, UK; 6 Department of Drug Discovery and Biomedical Sciences, Medical University of South Carolina, Charleston, SC USA; 7 School of Medicine, Faculty of Health Sciences and Wellbeing, University of Sunderland, Sunderland, UK

**Keywords:** Paediatric cancer, Targeted therapies

## Abstract

Deregulated polyamine biosynthesis is emerging as a common feature of neuroblastoma and drugs targeting this metabolic pathway such as DFMO are in clinical and preclinical development. The polyamine analog verlindamycin inhibits the polyamine biosynthesis pathway enzymes SMOX and PAOX, as well as the histone demethylase LSD1. Based on our previous research in acute myeloid leukemia (AML), we reasoned verlindamycin may also unblock neuroblastoma differentiation when combined with all-*trans*-retinoic acid (ATRA). Indeed, co-treatment with verlindamycin and ATRA strongly induced differentiation regardless of *MYCN* status, but in MYCN-expressing cells, protein levels were strongly diminished. This process was not transcriptionally regulated but was due to increased degradation of MYCN protein, at least in part via ubiquitin-independent, proteasome-dependent destruction. Here we report that verlindamycin effectively induces the expression of functional tumor suppressor—antizyme via ribosomal frameshifting. Consistent with previous results describing the function of antizyme, we found that verlindamycin treatment led to the selective targeting of ornithine decarboxylase (the rate-limiting enzyme for polyamine biosynthesis) as well as key oncoproteins, such as cyclin D and Aurora A kinase. Retinoid-based multimodal differentiation therapy is one of the few interventions that extends relapse-free survival in MYCN-associated high-risk neuroblastoma and these results point toward the potential use of verlindamycin in this regimen.

## Introduction

Neuroblastoma is the most common extra-cranial solid tumor in children. Half of the patients are diagnosed with high-risk disease, which requires multi-modal therapy including chemotherapy, surgery, and radiotherapy [1]. Despite intensive treatment, 5-year survival rate for this group of patients remains around 40%.

The two most important markers of poor survival probability in neuroblastoma are undifferentiated tumor morphology and MYC pathway activity [2]. Induction of differentiation is therefore one of the most promising approaches to treat neuroblastoma, with retinoic acid (RA) used in the clinic for patients with minimal residual disease [3]. However, many patients relapse while on retinoid treatment, which may suggest either gain of resistance or selection (and expansion) of a resistant clone. *MYCN* amplification, found in approximately 20% of neuroblastoma patients, indicates particularly bad prognosis [4]. MYCN transcription factor belongs to the MYC family and is involved in cell proliferation, growth, apoptosis and differentiation. Its expression is restricted mainly to embryonic development, which makes it a potentially good drug target [5]. However, so far direct MYCN inhibition (i.e., inhibition of MYCN binding to Max) has been rather challenging with no drugs available at the moment. MYCN is therefore considered to be undruggable due to reasons such as its nuclear localization and lack of a defined ligand-binding site [6, 7].

One way of indirect MYC targeting is via polyamine depletion [8, 9]. Polyamines are organic cations affecting numerous processes in tumorigenesis, such as cell proliferation, tumor growth, apoptosis, and angiogenesis [10]. There is a strong relationship between MYC and polyamine levels; one of the MYC target genes is ornithine decarboxylase (*ODC1*), a key enzyme in polyamine biosynthesis. Treatment with an ODC1 inhibitor—α-difluoromethylornithine (DFMO)—has been shown to decrease MYC levels [8]. DFMO is currently in phase II clinical trial for neuroblastoma patients in remission (NCT02395666, NCT01586260).

Besides inhibition of ODC1, polyamine synthesis can be also targeted via the use of polyamine analogs. One such compound is verlindamycin—a dual biguanide polyamine analog and LSD1 inhibitor (due to considerable homology between LSD1 and polyamine oxidases) [11]. Verlindamycin (also called compound 2d) was shown to inhibit growth of acute myeloid leukemia (AML) and estrogen-negative breast cancer in vitro [12, 13]. We previously demonstrated that LSD1 inhibition reactivates RA-induced differentiation in leukemia, which made us hypothesize that combination of verlindamycin and all-*trans*-retinoic acid (ATRA) can also be effective in neuroblastoma [14]. LSD1 is strongly upregulated in poorly differentiated neuroblastoma and its inhibition was shown to inhibit neuroblastoma growth both in vitro and in vivo [15]. In addition, polyamine depletion has been shown to induce differentiation in a mouse neuroblastoma cell line [16]. We therefore studied the impact of verlindamycin on neuroblastoma cells to show that this compound decreases proliferation rate and MYCN expression, whereas enhances cell differentiation in combination with ATRA. Taken together, we propose a novel mode of action of verlindamycin via upregulation of antizyme, a negative regulator of polyamine pathway.

## Materials and methods

### Compounds and cell culture

2d, 1,15–bis{N5–[3,3–(diphenyl)propyl]–N1–biguanido}–4,12–diazapentadecane, verlindamycin was generously provided by Dr Patrick Woster from Medical University of South Carolina; 10 mM stock in dimethyl sulfoxide (DMSO) was stored at −20 °C. ATRA (Sigma) was stored as a 10 mM stock in DMSO and ethanol (50/50) at −80 °C; GSK-LSD1 (Sigma) was stored at −20 °C as 1 mM stock solution in DMSO; DFMO (Enzo Life Sciences) was stored at −20 °C as 1 M solution dissolved in sterile water. Neuroblastoma cell lines were obtained from German Collection of Microorganisms and Cell Cultures (Kelly, SH-SY5Y), Public Health Collection (SK-N-BE(2)-C), American Type Cell Collection (SK-N-AS, IMR32, HEK-293T), and were all short tandem repeat-profiled. SHEP-Tet21/N were a gift from Dr Deborah Tweddle (Newcastle, UK). Cells were grown in RPMI-1640 medium (Sigma) supplemented with 10% fetal bovine serum (FBS; Gibco) without addition of antibiotics except for HEK-293T, which were grown in Dulbecco’s Modified Eagle Medium with 10% FBS and 1% PenStrep. Cells were cultured in standard conditions (5% CO_2_ and 37 °C) and routinely tested for *Mycoplasma* species (Sigma MP0035, LookOut® Mycoplasma PCR Detection Kit). For three-dimensional (3D) sphere culture, 2500 cells per well were seeded in ultra-low attachment round bottom plate (Costar, 7007) and imaged with Celigo to monitor growth. To establish 50% growth-inhibitory concentration (GI_50_) for verlindamycin, cells were grown in 96-well plates for 96 h and then treated with a range of concentrations. After 72 h of compound exposure, cells were fixed and cell viability was assessed with the use of sulforhodamine B assay [17]. GI_50_ was calculated with the use of GraphPad Prism.

### Cell proliferation assay and clonogenic assay

To assess difference in proliferation rate, cells were seeded in two black 96-well plates. Twenty-four hours after seeding, compounds were added to plates. The first plate was read as a reference after 24 h using CellTiter-Glo (Promega), according to the manufacturer’s protocol, on Synergy 2 Microplate Reader (BioTek). The second plate was read in the same way after 6 days of incubation. For clonogenic assay, cells were treated with compounds for 10 days after which they were seeded into 6-well plates, 1000 live cells per well. After 2 weeks of culture, cells were fixed with 4% paraformaldehyde and stained with crystal violet solution (Sigma). Colonies were counted with Oxford Optronics Gelcount.

### Annexin V flow cytometry assay

FITC annexin V apoptosis detection kit (BD Pharmingen) was used to assess apoptosis in cells treated with compounds for 6 days, according to the manufacturer’s protocol.

### Western blotting

Cells were lysed directly in LDS sample buffer with reducing agent (Invitrogen); samples were sonicated and denatured by 5 min incubation at 95 °C. Equal amount of protein was loaded on a gel and run at 130 V, then transferred to polyvinylidene difluoride membrane. Next, the membrane was blocked with 5% milk and incubated with primary antibody (Santa Cruz: MYCN Sc53993; Cell Signaling: glyceraldehyde 3-phosphate dehydrogenase (GAPDH) 2118L, Aurora A 4718, Cyclin D1 2922; Abcam: ODC1 ab97395, LSD1 ab17721) at 1:1000 for 2 h at room temperature. After washing, the membrane was incubated with secondary antibody conjugated with horseradish peroxidase (Dako) at 1:10,000 and washed again, and the reaction was developed with Lumigen ECL reagents (Lumigen) and imaged on LAS-3000 Imaging System (Fujifilm).

### Protein stability assays

For proteasomal inhibition, cells treated with verlindamycin for 4 days were treated with 10 μM MG-132 (Sigma) for 16 h. To assess MYCN half-life in cells treated with verlindamycin, cells were exposed to 25 μg/ml cycloheximide for up to 2 h. Cells were harvested, and after performing western blotting, band intensity was quantified with ImageJ.

### Immunofluorescence and proximity ligation assay (PLA)

Cells were grown on coverslips, treated with compounds, and fixed with 4% paraformaldehyde on day 6. Next, they were permeabilized with 0.2% Triton X-100 in phosphate-buffered saline (PBS), washed, and blocked for 30 min in immunofluorescence buffer (IFF; 1% bovine serum albumin, 2% FBS in PBS). Cells were then incubated with primary antibody (Santa Cruz: MYCN Sc53993 1:5000; Cell Signaling: Neurofilament-l 2837S 1:100) for 1 h at room temperature. After washing, cells were incubated with secondary antibody (goat anti-rabbit Alexa-Fluor 568 and goat anti-mouse Alexa-Fluor 488, Invitrogen) diluted 1:5000 in IFF for 1 h. Finally, they were washed with PBS (last wash with 1:5000 4,6-diamidino-2-phenylindole) and nanopure water and mounted in Fluoromount-G (SouthernBiotech). Fluorescence was observed with Zeiss confocal microscope, with the use of ZEN software. DuoLink PLA was performed according to the manufacturer’s protocol (Sigma); fluorescence was registered with Zeiss confocal microscope and scored with the DuoLink ImageTool software.

### RNA extraction, reverse transcription, and quantitative PCR (qPCR)

To analyze gene expression, cells were harvested at 70% confluency and total RNA was isolated (Zymo Research); 500 ng of RNA was used for reverse transcription (qScript, Quantas). For SYBR qPCR, 5 ng of cDNA was mixed with primers (*RARB* Fwd TGAGTCCTGGGCAAATCCTG Rev CGGTTTGGGTCAATCCACTGA, *CRABP2* Fwd TCGGAAAACTTCGAGGAATTGC Rev CCTGTTTGATCTCCACTGCTG, *TrkB* Fwd TGTTCAGCACATCAAGCGACA Rev CAAAGGCTCCTTCGCCTAGC, *Ret* Fwd GGCATCAACGTCCAGTACAAG Rev TGAGGTGACCACCCCTAGC, *GAPDH* Fwd ATGGGGAAGGTGAAGGTCG Rev TAAAAGCAGCCCTGGTGACC), master mix (SYBR Green PCR Master Mix), and water. For TaqMan qPCR, 5 ng of cDNA was mixed with Applied Biosystems probes (*MYCN* Hs00232074_m1, *OAZ1* Hs00427923_m1, *GAPDH* 4310884E), master mix (Gene Expression Master Mix, Life Technologies), and water. qPCR reaction was performed as follows: 50 °C for 2 min, 95 °C for 10 min, (95 °C for 15 s, 60 °C for 1 min) × 40 cycles on StepOnePlus Real-Time PCR Systems (Life technologies) with melt curve analysis for SYBR. Gene expression was normalized to GAPDH with comparative Ct method.

### Small interfering RNA (siRNA) transfection

siRNA targeting *OAZ1* and *LSD1* and non-targeting was prepared according to the manufacturer’s protocol (siGENOME SMART Pool, Dharmacon). Briefly, siRNAs and DharmaFECT (Dharmacon; 0.1% (v/v) of final volume) were added to separate tubes with serum-free media and incubated for 5 min at room temperature. After that, DharmaFECT was distributed between tubes with siRNAs and tubes were incubated at room temperature for 20 min. Afterwards, complete media (with or without verlindamycin) was added to tubes and distributed to each well. Knockdowns were observed after 96 h (and cells were re-treated after 72 h according to the same protocol where indicated).

### Expression microarray

To study the effect of combined treatment with verlindamycin and ATRA on gene expression in neuroblastoma cell lines, SK-N-BE(2)-C and SK-N-AS were treated with a combination of verlindamycin (1.75 μM = 0.5× GI_50_) and ATRA (1 μM). Cells were treated on day 0, re-treated on day 3, and harvested on day 6. RNA was extracted and sent to Oxford Gene Technology in Oxford to perform the Agilent expression microarrays and analysis. Genes with a false discovery rate (FDR) of 0.05 were selected as representing significant differential expression and the list was further filtered to include those where the logFC was either >1 or <−1. Expression of genes with logFC values between groups outside the range −1.5 to +1.5 (a subset of the most strongly differentially expressed genes) was then visualized as heatmaps. We also used the pathway commons 2 database to annotate differentially expressed genes with known regulatory relationships (“controls-expression-of”) to show networks of upregulated and downregulated nodes with edges representing regulatory relationships with the differentially expressed regulatory genes (nodes). Raw data, final, normalized results as well as microarray metadata has been deposited in NCBI’s gene expression omnibus as series GSE178900.

### Frameshifting assay

The assay was performed in HEK293T cell line; therefore, first verlindamycin GI_50_ was established in HEK293T. Cells were seeded in 96-well plates and treated with a range of compound concentrations. After 48 h of compound exposure, cell viability was assessed with CellTitre-Blue on Promega GloMax Discover plate reader. We received four plasmids from Dr John F. Atkins: *Oaz1* wild type, *oaz1* in frame control, *oaz2* wild type, and *oaz2* in frame control in a pSGCluc plasmid vector containing a dual luciferase reporter system (Loughran et al. [18]). Luciferase activity was observed in wild-type plasmids if frameshifting occurred, whereas in the control plasmids, no frameshifting was needed as a thymine nucleotide is missing from the DNA sequence coding for antizyme 1 and 2. For the frameshifting assay, HEK293T cells were seeded into white 96-well plates at a density of 20,000 cells/well with the addition of 2.5 mM DFMO. After 24 h, cells were transfected with 0.2 ng of plasmids with the use of Lipofectamine (Invitrogen). After 6 h transfection, solutions were removed and cells were treated with compounds (verlindamycin and spermidine) at an indicated concentration for 48 h. Afterwards, Dual-Glo Stop & Glo Luciferase Assay System (Promega) was used to analyze Firefly and Renilla luciferase activities and the luminescence was measured on GloMax Discover plate reader. Percentage frameshifting (%FS) activity was determined by obtaining firefly:renilla luciferase ratios, then dividing reporter values by in-frame control values. Relative FS was calculated by the following method: background %FS activity determined from the 2.5 mM DFMO control was subtracted from the %FS activity for the treated samples. The background-corrected %FS activity of each compound was then divided by the background-corrected %FS activity induced by 25 mM spermidine and multiplied by 100.

## Results

### Verlindamycin inhibits neuroblastoma cell proliferation and enhances the growth-inhibitory effect of ATRA

**Fig. 1:**
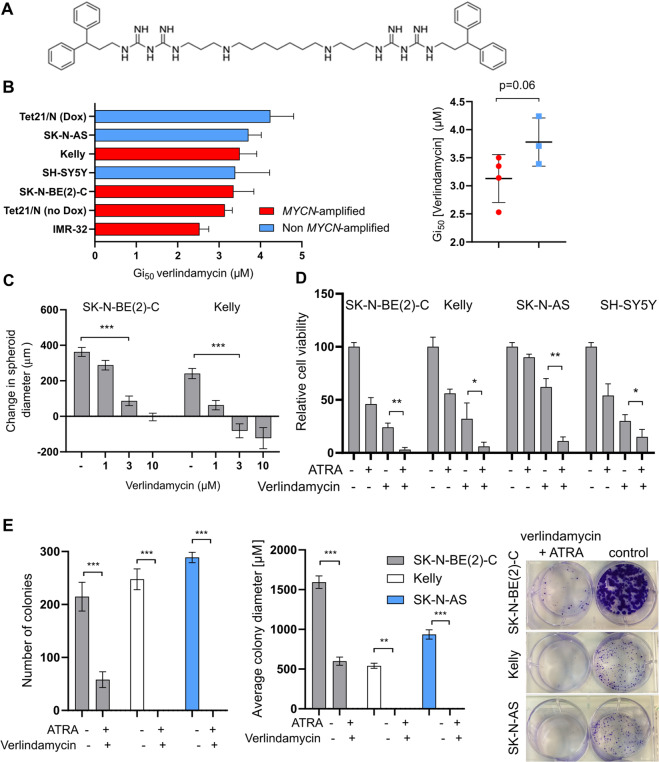
Verlindamycin inhibits neuroblastoma cell proliferation and enhances growth-inhibitory effect of ATRA. **A** Structure of verlindamycin. **B** Dose response by SRB assay for a panel of non *MYCN*-amplified (blue) and *MYCN*-amplified (red) neuroblastoma cell lines treated with verlindamycin for 72 h. 50% growth-inhibitory (GI_50_) values are shown for individual cell lines (left panel) and non-*MYCN*-amplified versus *MYCN*-amplified cell lines (right panel). **C** SK-N-BE(2)-C and Kelly cells cultured as tumor spheroids were treated with verlindamycin for 72 h and changes in spheroid diameter were assessed using a Celigo S Imaging Cell Cytometer. **D** Viability assessment as measured by CellTiter Glo (Promega) in SK-N-BE(2)-C and Kelly cells grown in monolayer. Metabolic activity of cells treated with 0.5× GI_50_ verlindamycin in combination with 1 μM ATRA was measured by CellTiter Glo after 6 days of treatment. **E** Cells treated with 0.5× GI_50_ verlindamycin and 1 μM ATRA for 10 days were tested for their ability to form colonies from single cells within 14 days. All the experiments were performed in triplicates and a representative result or mean is shown. Student’s *t* test was performed to calculate statistical significance, *p < 0.05, **p < 0.005, ***p < 0.0005, error bars show standard deviation.

In order to analyze the effects of verlindamycin (Fig. 1A), we first performed dose–response assays in a panel of neuroblastoma cell lines. Verlindamycin inhibited growth in all neuroblastoma cell lines tested with GI_50_ values in the micromolar range (Fig. 1B and Supplementary Fig. 1). *MYCN*-amplified cells were more sensitive to the compound compared with non *MYCN*-amplified cells, but the difference was not statistically significant. Next, we tested verlindamycin efficiency on tumor spheroids—neuroblastoma cells grown in 3D—as this type of cell culture provides a better representation of tumor physiology than cells grown in monolayer. We found that verlindamycin treatment decreased growth of tumor spheroids (as measured by spheroid diameter change, which was from −4 for 10 μM to +363 μm for control in Kelly and from −123 μm for 10 μM to +241 μm for control in SK-N-BE(2)-C) with GI_50_ values similar to those obtained with adherent cells (3.35 μM in two-dimensional (2D) versus 4.7 μM in 3D for SK-N-BE(2)-C and 3.5 μM in 2D versus 1.58 μM in 3D for Kelly) (Fig. 1C). Treatment with either verlindamycin or ATRA alone for 6 days decreased the proliferation rate of neuroblastoma cells; however, the combined treatment had the strongest antiproliferative effect leaving only 2–15% of cells metabolically active (Fig. 1D).

Because the treatment with ATRA and verlindamycin left very few metabolically active cells, we hypothesized that it may induce cell death. To test this hypothesis, cells were stained with annexin V (Supplementary Fig. 2), which revealed that verlindamycin and ATRA treatment increases percentage of cells both in early and late apoptosis (SK-N-BE(2)-C: 16% of apoptotic cells in control versus 50% in treated; Kelly: 7 versus 82%; SKNAS: 7 versus 41%). To address the question whether cells that survive verlindamycin and ATRA treatment would be able to grow at low cell density, we performed a clonogenic assay. Cells treated with verlindamycin and ATRA for 10 days (alongside controls) were seeded at 1000 cells per well and cultured for 2 weeks. Cells treated with verlindamycin and ATRA were not only forming significantly less colonies, if any, but the colonies were also smaller (Fig. 1E). Thus, treatment with the combination of verlindamycin and ATRA leads to decrease in cell proliferation and induction of cell death in both 2D and 3D cultures.

### Verlindamycin enhances RA-induced differentiation in neuroblastoma

**Fig. 2:**
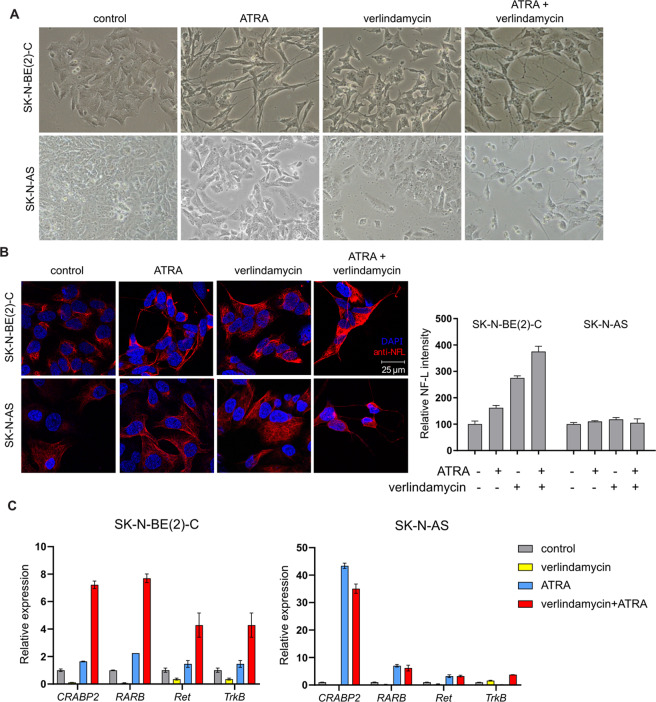
Verlindamycin enhances ATRA-induced differentiation in neuroblastoma. SK-N-BE(2)-C and SK-N-AS were treated with 0.5× GI_50_ verlindamycin and 1 μM ATRA for 6 days. **A** Brightfield microscopic pictures show morphological differences upon treatment. **B** Cells were fixed and stained with neurofilament light chain (NFL, red) and DAPI (blue). **C** mRNA expression of ATRA-target gene (*CRABP2*) and neural markers (*RARB*, *RET*) was measured by RT-qPCR and normalized to *GAPDH*. All the experiments were performed in triplicates and a representative result or mean is shown; error bars show standard deviation.

After establishing that verlindamycin has an antiproliferative effect on its own and that it enhances the growth-inhibitory effect of ATRA, we investigated whether its cytostatic effect may be caused by cell differentiation. After 6 days of combined treatment, some profound changes in cell morphology could be observed, with cells elongating and extending neurites (Fig. 2A and Supplementary Fig. 3A). Staining with neurofilament-light, a well-established neural marker, confirmed that the cell extensions observed by a light microscope are neurites (Fig. 2B and Supplementary Fig. 3B). In *MYCN*-amplified SK-N-BE(2)-C and Kelly, the intensity of staining and the number of neurites were increased by both ATRA and verlindamycin single treatments, with the strongest effect in the combined treatment. Interestingly, the combined verlindamycin and ATRA treatment led to a modest induction of neurites also in the non-neuronal SK-N-AS. To confirm differentiated status of cells treated with verlindamycin and ATRA, we assessed the expression of a set of previously published RA-response genes: *CRABP2* and *RARB*, and neural markers: *RET* and *TrkB* [19]. Remarkably, markers’ levels in cells treated with combination of compounds were higher than in cells treated with any of the agents alone, especially in *MYCN*-amplified cells such as SK-N-BE(2)-C and Kelly (Fig. 2C and Supplementary Fig. 3C). This indicates that there may be a synergistic effect between verlindamycin and ATRA, possibly down to the epigenetic role of verlindamycin. Overall, these results indicate that addition of verlindamycin enhances the differentiation-inducing properties of ATRA.

**Fig. 3:**
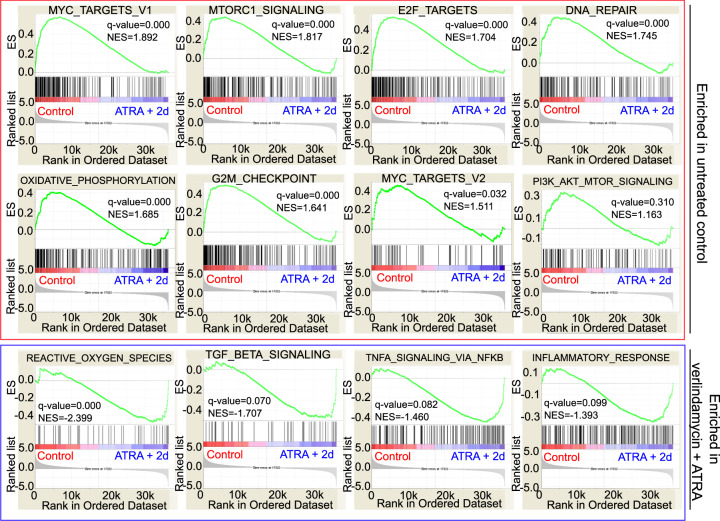
Gene Set Enrichment Analysis (GSEA) of differential gene expression in SK-N-BE(2)-C cells following co-treatment with verlindamycin (2d) and ATRA. Gene expression data generated by expression microarray analysis following 6 days of co-treatment with 1 μM ATRA and 0.5× Gi_50_ verlindamycin versus untreated (DMSO) control were analyzed using GSEA to extract biological knowledge and highly significantly enriched gene sets are shown. The most upregulated genes in vehicle control are shown on the left side (red), while the most upregulated genes following ATRA + 2d treatment are shown on the right side (blue). Black bars represent the positions of the vehicle control versus ATRA + 2d upregulated signature genes in the ranked list. Green curves represent the evolution gene density. Normalized enrichment scores (NES) reflect the degree to which genes are overrepresented. When the distribution is random, the enrichment score is zero. Enrichment of signature genes at the top of the ranked list results in a large positive deviation of the NES from zero. *q*-value false discovery rate (FDR)-adjusted *q*-value.

To assess the underlying effect of verlindamycin and ATRA treatment on neuroblastoma cells, we performed transcriptomics analysis of *MYCN*-amplified SK-N-BE(2)-C and non-*MYCN*-amplified SK-N-AS. Expression microarrays were performed after 6 days of treatment—on cells that had inhibited growth and were well differentiated. In both cell lines, we noted that histograms of adjusted *p* values (FDR) had non-uniform distributions that included a sharp peak of very small adjusted *p* values, in contrast to the uniform distribution of *p* values expected with random data (Supplementary Fig. 4A, D). Differential gene expression analysis between the ATRA plus verlindamycin-treated and vehicle-treated SK-N-BE(2)-C cells identified 348 spots as significantly upregulated, corresponding to 255 unique genes, and a further 539 significantly downregulated spots, corresponding to 378 unique genes. In a similar analysis of the SKNAS gene expression data set, a total of 952 spots showed increased expression corresponding to 661 unique genes, while 600 spots showed downregulation, corresponding to 427 unique genes. Volcano plots revealed that a small number of spots represented both highly significant and strongly differentially expressed genes and they were not restricted to either highly expressed or weakly expressed genes (Supplementary Fig. 4B, C, E, F). We next visualized the expression of a subset of the most strongly differentially expressed genes in each group as heatmaps (Supplementary Fig. 5). Supplementary Fig. 6 shows networks of upregulated (yellow) and downregulated (cyan) nodes with edges representing regulatory relationships with the differentially expressed regulatory genes (gray nodes). Gene Set Enrichment Analysis revealed downregulation of several oncogenic pathways, such as mammalian target of rapamycin (mTOR), phosphoinositide-3 kinase (PI3K)-Akt, and DNA repair (Fig. 3 and Supplementary Fig. 7). Additionally, MYC targets were highly upregulated in control compared to verlindamycin- and ATRA-treated cells. Taken together, these results suggest that reduced proliferation of cells treated with verlindamycin and ATRA may be due to downregulation of several oncogenic pathways.

### Verlindamycin downregulates the expression of MYCN protein by targeting its stability

**Fig. 4:**
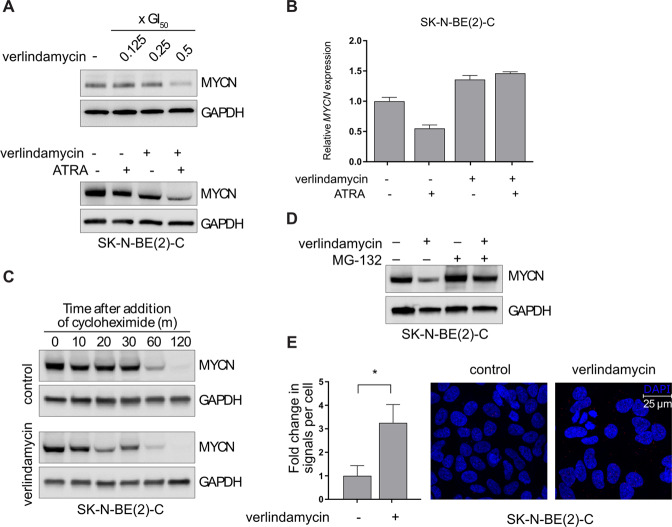
Verlindamycin downregulates the expression of MYCN protein in *MYCN*-amplified neuroblastoma. **A** MYCN protein levels were assessed in SK-N-BE(2)-C cells treated for 6 days: with different concentrations of verlindamycin (upper panel; GI_50_ = 3.5 μM) and with 0.5× GI_50_ verlindamycin combined with 1 μM ATRA (lower panel). **B** *MYCN* mRNA expression was measured by RT-qPCR (relative to *GAPDH*) in SK-N-BE(2)-C cells treated for 6 days with 0.5× GI_50_ verlindamycin alone or combined with 1 μM ATRA. **C** SK-N-BE(2)-C cells pretreated with 0.5× GI_50_ verlindamycin for 4 days were exposed to 25 µg/ml cycloheximide for up to 2 h. **D** SK-N-BE(2)-C cells pretreated with verlindamycin for 4 days were exposed to 10 µM MG-132 for 16 h. **E** PLA was performed on SK-N-BE(2)-C to assess MYCN-FBXW7 interaction; dots were quantified (left panel). All the experiments were performed in triplicates and a representative result or mean is shown; error bars show standard deviation. Student’s *t* test was performed to calculate statistical significance, *p < 0.05.

MYC targets pathway was prominently downregulated upon verlindamycin and ATRA treatment (Fig. 3). MYCN is a major determinant of outcome in neuroblastoma, amplified in the most aggressive tumors. No direct MYCN inhibitor is currently available while a lot of research interest is focused on the development of therapeutics that target MYCN indirectly [20–22]. As *MYCN*-amplified and *MYCN*-driven neuroblastoma cell lines were very sensitive to the combination of verlindamycin and ATRA, we investigated the effect of this combined treatment on MYCN itself. We measured the expression of MYCN protein upon treatment with increasing levels of verlindamycin and its combination with ATRA. Expression of MYCN protein was affected by as little as 1.75 µM (0.5× GI_50_) of verlindamycin and the effect was even stronger upon addition of ATRA (Fig. 4A and Supplementary Fig. 8A). Interestingly, expression of *MYCN* mRNA in SK-N-BE(2)-C and Kelly cells treated with verlindamycin and ATRA for 6 days was not downregulated (Fig. 4B and Supplementary Fig. 8B). Therefore, we hypothesized that this treatment targets MYCN protein but not the transcription of its mRNA. To test this hypothesis, we examined the effect of verlindamycin on MYCN stability and proteasomal degradation. Cells treated with verlindamycin were exposed to cycloheximide (an inhibitor blocking protein synthesis) for 2 h. We found that verlindamycin decreases ~2-fold MYCN half-life (20 min for treated versus 50 min for untreated in SK-N-BE(2)-C and 25 versus 60 min in Kelly) (Fig. 4C and Supplementary Fig. 8C). We then tested whether MYCN protein levels are reduced because of protein degradation by the proteasome. We found that treatment with the proteasomal inhibitor MG-132 brings the level of MYCN in verlindamycin-treated cells back to control level (Fig. 4D and Supplementary Fig. 8D). We then performed PLA to study the MYCN–Fbxw7 interaction in cells treated with verlindamycin (Fig. 4E). FBXW7 ubiquitin ligase interacts with MYCN to target it for proteasomal degradation, and as we expected, cells treated with verlindamycin had significantly more FBXW7-MYCN complexes. All these data suggest that verlindamycin downregulates the expression of MYCN by targeting its stability. To further study the connection between MYCN levels and cell differentiation, we also investigated the effect of direct MYCN inhibition by siRNA on neuroblastoma cells. We found that MYCN downregulation by siRNA leads to neuroblastoma differentiation as measured by morphological changes and NF-L-expressing neurite outgrowth (Supplementary Fig. 9), which may indicate that differentiated cells should have lower MYCN levels.

### Verlindamycin is not acting via inhibition of LSD1

**Fig. 5:**
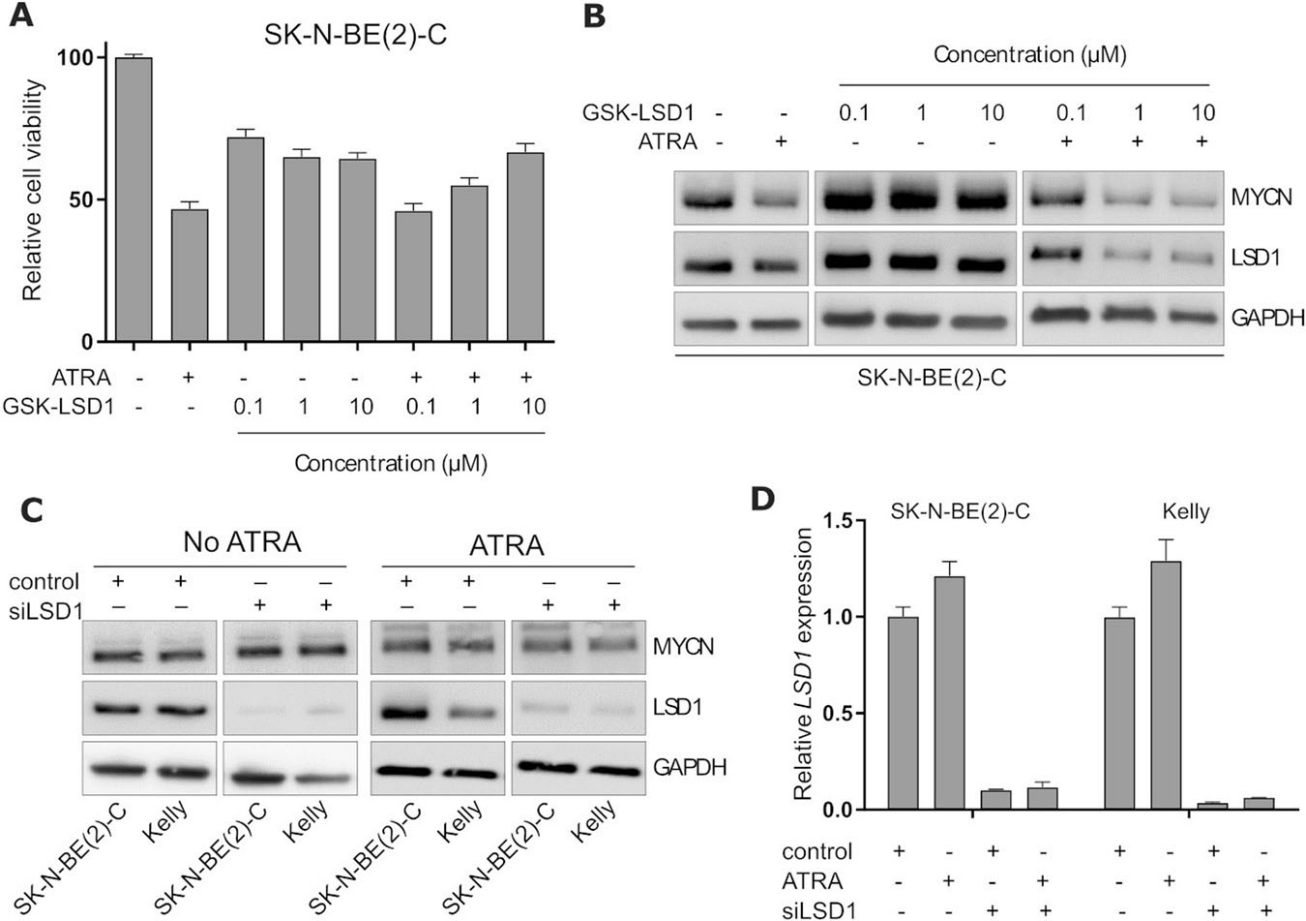
Inhibition of KDM1A/LSD1 is not the mechanism of action of verlindamycin. **A** SK-N-BE(2)-C cells were treated with a range of GSK-LSD1 concentrations combined with 1 μM ATRA for 6 days after which cell viability was assessed by CellTiter Glo. **B** MYCN expression level was tested in cells treated with GSK-LSD1 with or without addition of 1 μM ATRA for 6 days. **C** SK-N-BE(2)-C and Kelly cells with siRNA-mediated *KDM1A* knock-down (alongside non-targeting control) and treated with ATRA (for 6 days) were harvested to assess the expression of MYCN and LSD1. **D** mRNA expression of *KDM1A* was also tested in SK-N-BE(2)-C and Kelly cells treated with ATRA and *KDM1A*-targeting siRNA; expression measured by RT-qPCR relative to *GAPDH*. All the experiments were performed in triplicates and a representative result or mean is shown; error bars show standard deviation.

Our results show that verlindamycin is a potent inhibitor of neuroblastoma growth and an enhancer of ATRA-induced differentiation. Because this compound is a dual LSD1 inhibitor and polyamine analog, we decided to test whether it is acting via inhibition of LSD1. We first tested GSK-LSD1, recently described as a potent LSD1 inhibitor in small cell lung cancer [23]. We treated neuroblastoma cells with a range of GSK-LSD1 concentrations with or without the addition of ATRA (Fig. 5A and Supplementary Fig. 10). GSK-LSD1 had a modest growth-inhibitory effect and was not amplified by ATRA. In addition, GSK-LSD1 did not downregulate the expression of MYCN protein (Fig. 5B and Supplementary Fig. 10). To ensure that LSD1 inhibition is not the mechanism of action of verlindamycin, we also tested a genetic blocker. Even though the siRNA-mediated knockdown of LSD1 (encoded by *KDM1A* gene) was very efficient (protein level in Fig. 5C and RNA in Fig. 5D), it did not lead to MYCN downregulation. Therefore, all the evidences show that the growth-inhibitory and MYCN downregulating effect of verlindamycin is not due to LSD1 inhibition.

### Verlindamycin induces upregulation of antizyme and leads to downregulation of antizyme targets

**Fig. 6:**
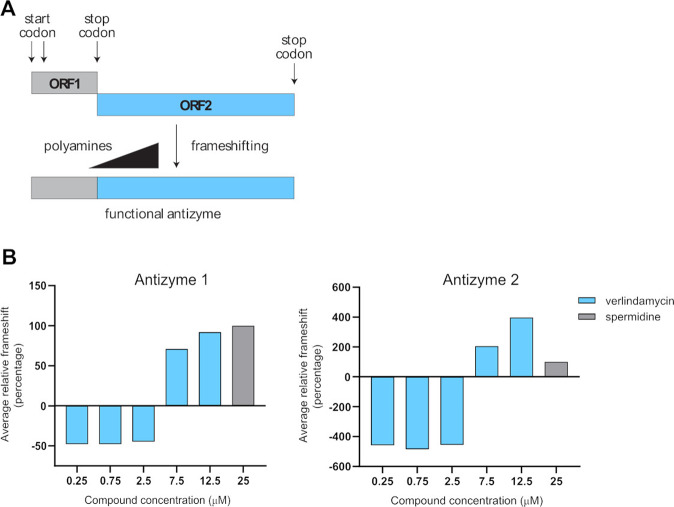
Verlindamycin treatment induces frameshifting of antizymes 1 and 2. **A** Schematic showing polyamine-induced frameshifting of antizyme. **B** Verlindamycin-induced frameshifting of antizymes 1 and 2 relative to 25 µM spermidine. Briefly, 293T cells were transfected with antizyme 1 or 2 frameshifting reporters or in-frame controls with the addition of 25 µM spermidine or verlindamycin as indicated. Percentage frameshifting (%FS) activity was determined with Dual-Glo (Promega) by obtaining firefly:renilla luciferase ratios, then dividing reporter values by in-frame control values. The background-corrected %FS activity of each compound concentration was then divided by the background-corrected %FS activity induced by 25 mM spermidine and multiplied by 100. All the experiments were performed in triplicates and a representative result is shown.

It was reported that increase in polyamine levels leads to downregulation of ODC1 as a feedback mechanism to regulate and keep polyamine levels low [24]. This process is triggered by antizyme—a protein that leads to ubiquitin-independent degradation of ODC1. The schematic showing the frameshifting of antizyme upon polyamine induction is depicted in Fig. 6A. To assess whether the increase in polyamine analog levels will also upregulate functional antizyme levels, we performed frameshifting assay using Dual Luciferase Reporter System (as previously described [18]). For frameshifting assay, HEK293 cells were transfected with wild-type or in-frame control plasmids for antizyme 1 and 2. Verlindamycin was added on to cells alongside spermidine (as a positive control) and the assay was read with the use of Dual-Glo Stop & Glo Luciferase Assay System with the results shown in Fig. 6B. Even though the cells were treated with verlindamycin for 24 h only, we already observed a strong induction of antizyme at GI_50_ concentrations (GI_50_ presented in Supplementary Fig. 1). These results indeed indicate that verlindamycin induces antizyme frameshifting.

**Fig. 7:**
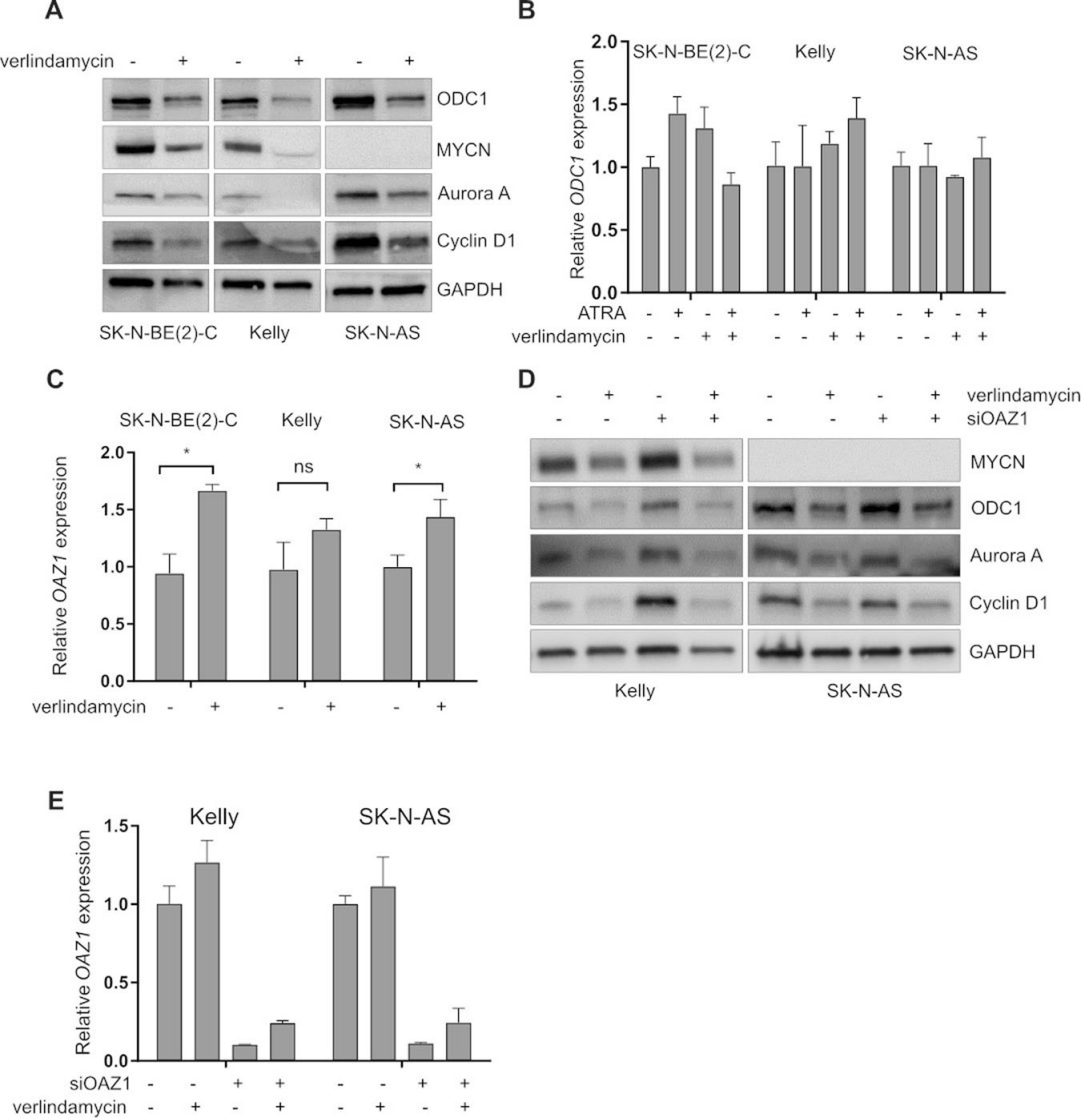
Functional analysis of verlindamycin-induced frameshifting of antizyme. **A** SK-N-BE(2)-C, Kelly, and SK-N-AS cells were treated with 0.5× GI_50_ verlindamycin for 4 days, after which the expression of Cyclin D1, Aurora A, and MYCN was measured. GAPDH expression is shown as a loading control. At the same time, the expression of **B** *ODC1* and **C** *OAZ1* mRNA was measured by qPCR, shown relative to GAPDH. **D**, **E** After 96 h of siRNA knock-down of *OAZ1* combined with verlindamycin treatment, **D** the expression of OAZ1 targets was measured by western blotting. **E** Expression of *OAZ1* mRNA was assessed by RT-qPCR (relative to *GAPDH*) in cells transfected with siRNA. All the experiments were performed in triplicates and a representative result or mean is shown; error bars show standard deviation. Student’s *t* test was performed to calculate statistical significance, *p < 0.05.

We then further assessed whether the induced antizyme was able to downregulate its targets in neuroblastoma. Four-day verlindamycin treatment led to significant ODC1 downregulation (Fig. 7A) without affecting its transcription (Fig. 7B). Simultaneously, other known targets of antizyme—cyclin D1 and Aurora A—were also downregulated at a protein level. In *MYCN*-amplified cells (Kelly and SK-N-BE(2)-C), downregulation of Aurora A was coupled with a decrease in MYCN level. At the same time, we also showed that mRNA level of *OAZ1* increased upon verlindamycin treatment in all tested cell lines (Fig. 7C). Next, we performed antizyme 1 (*OAZ1*) knockdown with siRNA. After 4 days of siRNA treatment (with or without verlindamycin), mRNA levels of *OAZ1* were measured alongside with antizyme targets (Fig. 7D, E). Even though mRNA levels of *OAZ1* were low upon combination of *OAZ1* knockdown and verlindamycin, ODC1, Aurora A, and cyclin D levels were at a level similar to verlindamycin-only treatment. This may suggest that antizyme has a long half-life and therefore siRNA knockdown cannot efficiently decrease protein levels or verlindamycin is counteracting the knockdown effect.

**Fig. 8:**
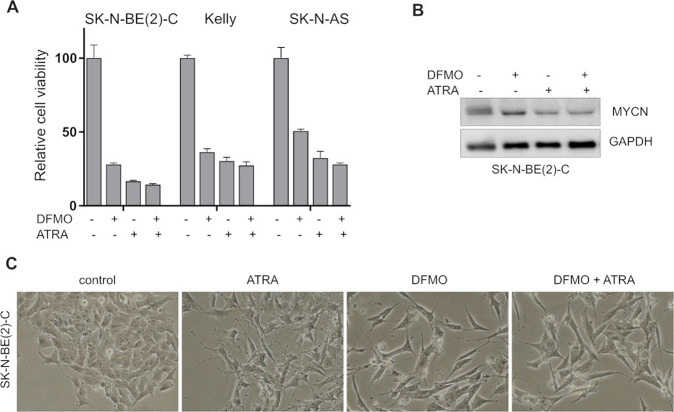
DFMO affects neuroblastoma viability and MYCN expression but does not enhance differentiation-inducing potential of ATRA. SK-N-BE(2)-C, Kelly, and SK-N-AS cells were treated with 5 mM DFMO, a well-studied ODC1 inhibitor, combined with 1 μM ATRA for 6 days. **A** Relative viability of those cells was measured by CellTiter Glo. **B** MYCN protein level was assessed in SK-N-BE(2)-C. **C** Cell morphology was observed via brightfield microscope. All the experiments were performed in triplicates and a representative result or mean is shown; error bars show standard deviation.

Finally, we compared verlindamycin to DFMO (eflornithine), an established ODC1 inhibitor. DFMO efficiently inhibited the growth of SK-N-BE(2)-C (Fig. 8A) but had a modest impact on MYCN expression (Fig. 8B). Additionally, 6-day treatment with DFMO did not enhance ATRA-induced differentiation (Fig. 8C). Therefore, DFMO did not recapitulate the effect of verlindamycin. All these results suggest that verlindamycin has a unique ability to induce antizyme, which degrades ODC1, Aurora A, and Cyclin D1 leading to reduced cell growth and MYCN expression.

## Discussion

In this study, we present a unique compound of dual potency—a polyamine analog as well as a LSD1 inhibitor, combined with an eminent differentiating agent—RA. We show that the addition of verlindamycin increases ATRA-driven differentiation (in neuroblastoma cell lines), indicated with distinctive morphology and expression of neural markers. RA has long been used as differentiation-inducing agent in the treatment of neuroblastoma and maximal morphological differentiation is usually reached after 6 days of RA exposure [25], which is a timepoint we used in this study. In clinical practice, 13-*cis* RA is the form of RA given to neuroblastoma patients as it has higher maximally tolerated dose and longer half-life than ATRA. However, 13-*cis* RA is isomerized to ATRA inside cells, which justified the use of ATRA in our in vitro experiments [26].

The growth of cells treated with the combination of verlindamycin and ATRA is almost completely inhibited and a big proportion of the cells became apoptotic. Verlindamycin alone induced apoptosis in Kelly cells, but to induce apoptosis in SK-N-BE(2)-C, it had to be combined with ATRA. Although both those cell lines are *MYCN* amplified, Kelly is derived from primary neuroblastoma while SK-N-BE(2)-C from metastatic site (bone marrow); therefore, more aggressive SK-N-BE(2)-C may be more resistant to verlindamycin-induced apoptosis. However, the level of differentiation induced by the combination of verlindamycin and ATRA was similar.

The growth-inhibitory effect of verlindamycin may be caused by polyamine depletion per se and/or by downregulation of MYCN, an oncoprotein, which is the primary driver of proliferation in the high-risk neuroblastoma subset. We show that verlindamycin is affecting MYCN stability and degradation but has no impact on its transcription. The mechanism behind it may be the upregulation of antizyme, a negative modulator of polyamine biosynthesis. Polyamines play a crucial role in cell growth and proliferation such that their levels have to be tightly controlled. High intracellular polyamine levels induce a ribosomal frameshift, which leads to production of a full-length antizyme protein [27]. Antizyme decreases polyamine levels in three different ways: by disrupting ODC1 dimers, by targeting ODC1 for degradation, and by inhibiting extracellular polyamine uptake. It was shown before that polyamine analogs stimulate cells for antizyme production and that antizyme levels correlate with growth inhibition [28]. Interestingly, ODC1 is not the only antizyme target, the other two being cell cycle regulatory protein cyclin D1 and Aurora-A, a key factor in regulating MYCN stability [29, 30]. Our results demonstrate increased antizyme frameshifting as well as degradation of antizyme targets (ODC1, Aurora A, Cyclin D1) upon treatment with verlindamycin. DFMO treatment did not recapitulate the effect of verlindamycin possibly because it has a different mode of action—through decreasing polyamine levels via direct ODC1 inhibition [31].

To understand deeper the mechanism underlying the profound effect of combined verlindamycin and ATRA treatment on neuroblastoma cells, we performed transcriptomics analysis. Even though *MYCN* mRNA was not downregulated by the treatment in SK-N-BE(2)-C, MYC targets were among the most strongly affected gene sets. This, however, was in line with our finding that there is a decrease in MYCN protein level due to increased MYCN degradation.

We have previously shown that PI3K/mTOR inhibitors kill *MYCN*-amplified neuroblastoma cells and have the ability to eliminate MYCN protein in vivo [32]. Verlindamycin appears to affect mTOR and PI3K pathways in both *MYCN*-amplified SK-N-BE(2)-C and non-*MYCN*-amplified SK-N-AS. Alterations in the PI3K pathway are frequent in tumors and promote sustained growth and proliferation [33]. Although the interaction between PI3K-mTOR pathway and polyamines has not been widely studied, it appears that polyamine production is increased upon PI3K activation [34]. Additionally, in prostate cancer mTORC1 has been recently shown to activate polyamine synthesis through the regulation of the enzymes involved in polyamine production [35]. mTORC1 can elevate polyamine levels by increasing ODC1 mRNA stability, and it also blocks antizyme production [36, 37]. We show that verlindamycin, as a polyamine analog, decreases polyamine levels and induces antizyme production leading also to a downregulation of PI3K-mTOR pathway in neuroblastoma.

Previously, verlindamycin has been mostly studied as an LSD1 inhibitor, not polyamine analog. The compound was selected in a library screen as the biguanide most efficiently inhibited the enzymatic activity of LSD1 [11]. Verlindamycin was shown to re-activate expression of aberrantly silenced genes in colon cancer, breast cancer, and AML cell lines [11–13]. We previously demonstrated that LSD1 inhibition with tranylcypromine reactivates ATRA-induced differentiation in AML, which then led to a promising clinical trial [14, 38]. MYCN directly interacts with LSD1 and inhibition of both MYCN and LSD1 was shown to be an efficient strategy for inhibition of neuroblastoma cell proliferation [39]. This may be why verlindamycin proved to be so efficient in neuroblastoma—it is a single molecule able to inhibit LSD1 and downregulate MYCN. However, because the effect of verlindamycin was not recapitulated by neither chemical nor genetic LSD1 inhibitor, we suggest that LSD1 blockade is not enough to enhance the differentiation-inducing potential of ATRA in neuroblastoma.

In conclusion, we hereby present this novel inhibitory mechanism of verlindamycin as a potential therapeutic treatment of neuroblastoma, including *MYCN*-amplified high-risk subgroup, for the first time.

## Supplementary information

Supplementary figures
