# Supplementary material for: The biguanide polyamine analog verlindamycin promotes differentiation in neuroblastoma via induction of antizyme
Source: Cancer Gene Ther. 2021 Sep 14;29(7):940–50. doi: 10.1038/s41417-021-00386-6 (PMC9293756; doi:10.1038/s41417-021-00386-6)
Supplement: Supplementary file 1 — Supplementary figures [file 41417_2021_386_MOESM1_ESM.docx]

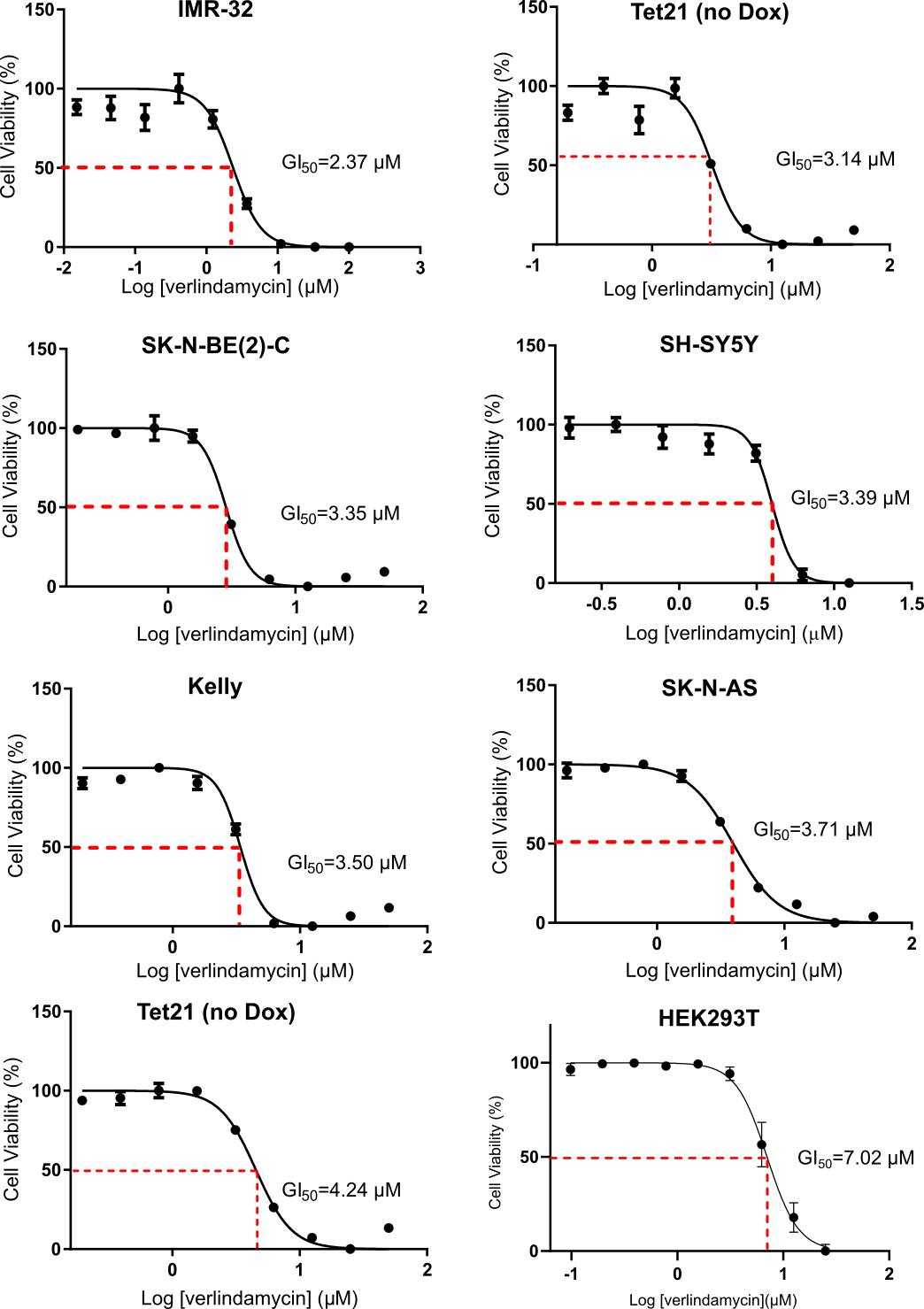


**Figure S1**. **GI_50_ curves of cells treated with verlindamycin.** Cells were treated with a range of drug concentration for 72h after which SRB assay was performed and GI_50_ curves were plotted with GraphPad Prism.


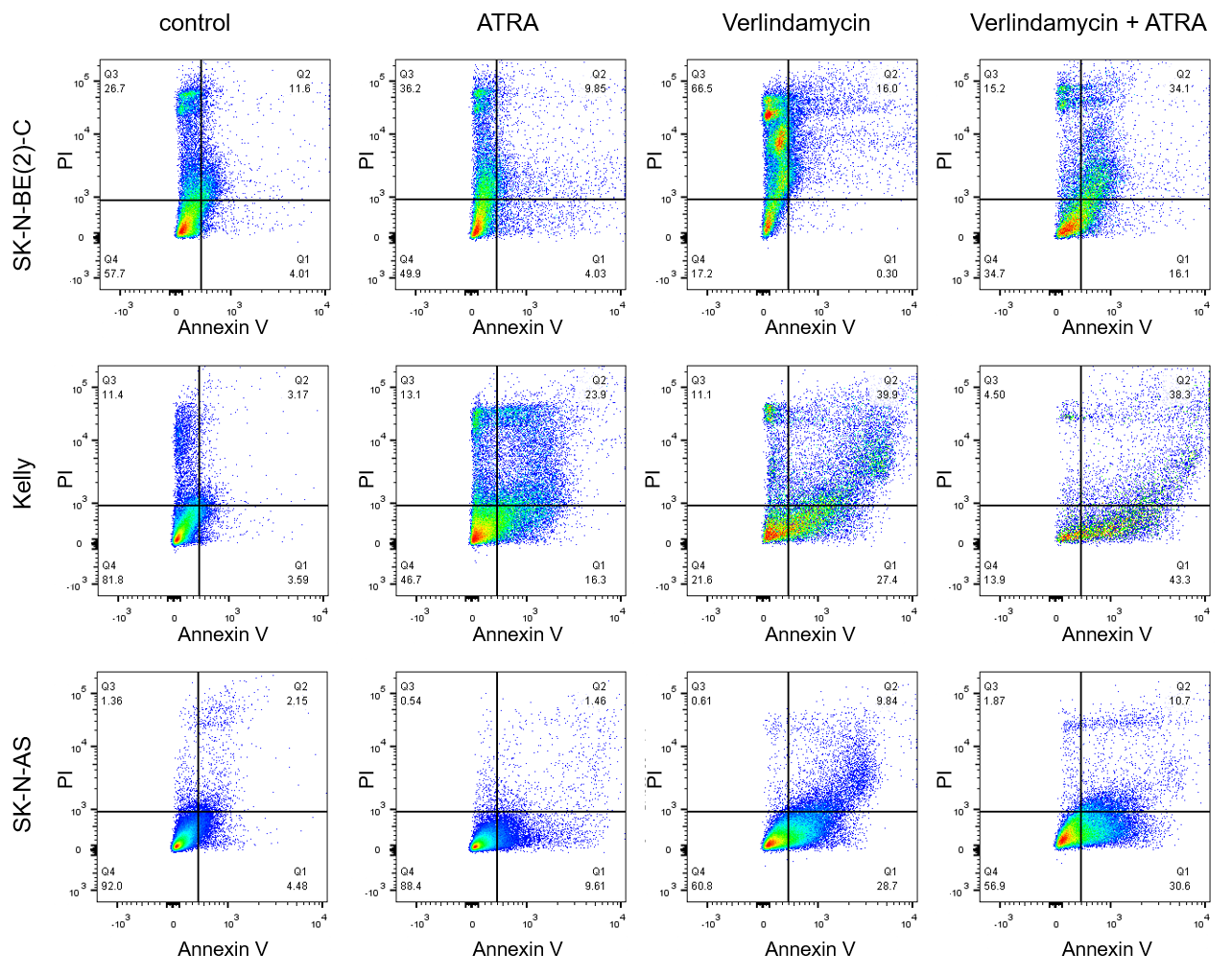


**Figure S2** **Verlindamycin treatment leads to increased apoptosis and cell death in neuroblastoma cell lines.** SK-N-BE(2)-C, Kelly and SK-N-AS were treated with 0.5 x GI_50_ verlindamycin and 1 μM ATRA for 6 days, fixed and stained with Annexin V FITC-conjugated antibody and PI to detect cell death. Treatment leads to increase in both early apoptotic cells (Q1) and late apoptotic or necrotic cells (Q2).


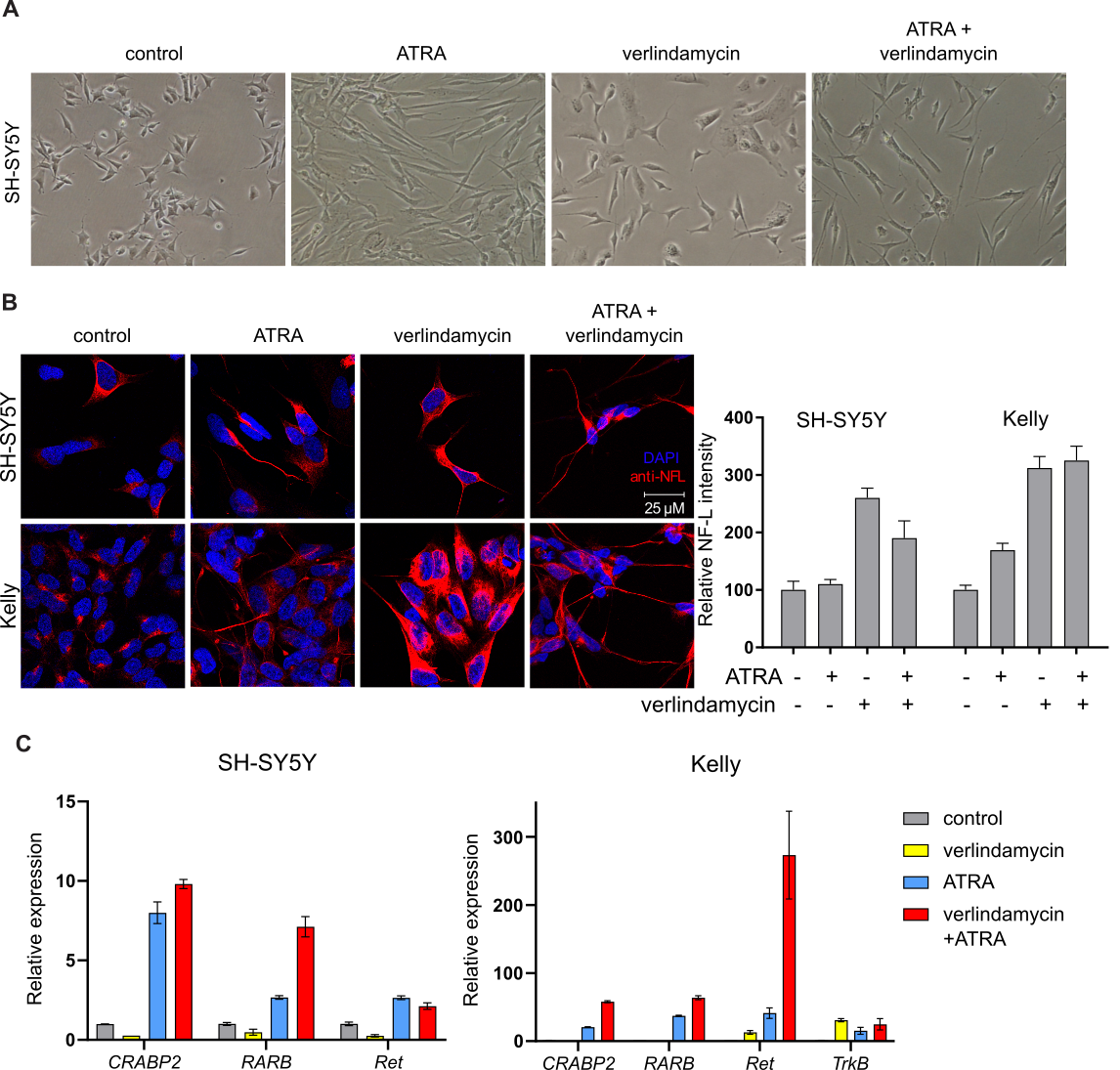


**Figure S3. Verlindamycin enhances ATRA-induced differentiation in neuroblastoma.** SH-SY5Y and Kelly were treated with 0.5 x GI_50_ verlindamycin and 1 μM ATRA for 6 days. (**A**) Brightfield microscope pictures show morphological differences upon treatment. (**B**) Cells were fixed and stained with neurofilament light chain (NFL, red) and DAPI (blue). (**C**) mRNA expression of ATRA-target genes (*CRABP2, RARB*) and neural markers (*Ret, TrkB*) was measured by RT-qPCR and normalized to *GAPDH.*

**Figure S4.** Summary of statistical analysis of differential gene expression in SK-N-BE(2)-C and SK-N-AS cell lines treated with ATRA and verlindamycin. (**A**) histogram of adjusted p-values (FDR) from all tests comparing treatments in SK-N-BE(2)-C cell lines. (**B**) Volcano plot summarising the significance and magnitude of changes following treatment of SK-N-BE(2)-C cell lines with ATRA and verlindamycin. (**C**) Scatter plot showing the average expression of genes with significant differences in expression (red points) in response to treatment of SK-N-BE(2)-C cell lines with ATRA and verlindamycin. (**D**), (**E**), (**F**) Equivalent plots of (**A**), (**B**), (**C**) but showing results for the SK-N-AS cell line.

**A**

**B**

**C**

**Figure S5.** (**A**) Heatmap showing the centred and scaled expression values of genes with significant differences between ATRA and verlindamycin treated (right three columns) versus control (left three columns) SK-N-BE(2)-C cells. (**B**) Equivalent heatmap to that shown in G representing data for SK-N-AS cells. (**C**) Scatter plot of genes with significantly different expression levels in both the SK-N-BE(2)-C and SK-N-AS cell lines treated with ATRA and verlindamycin.


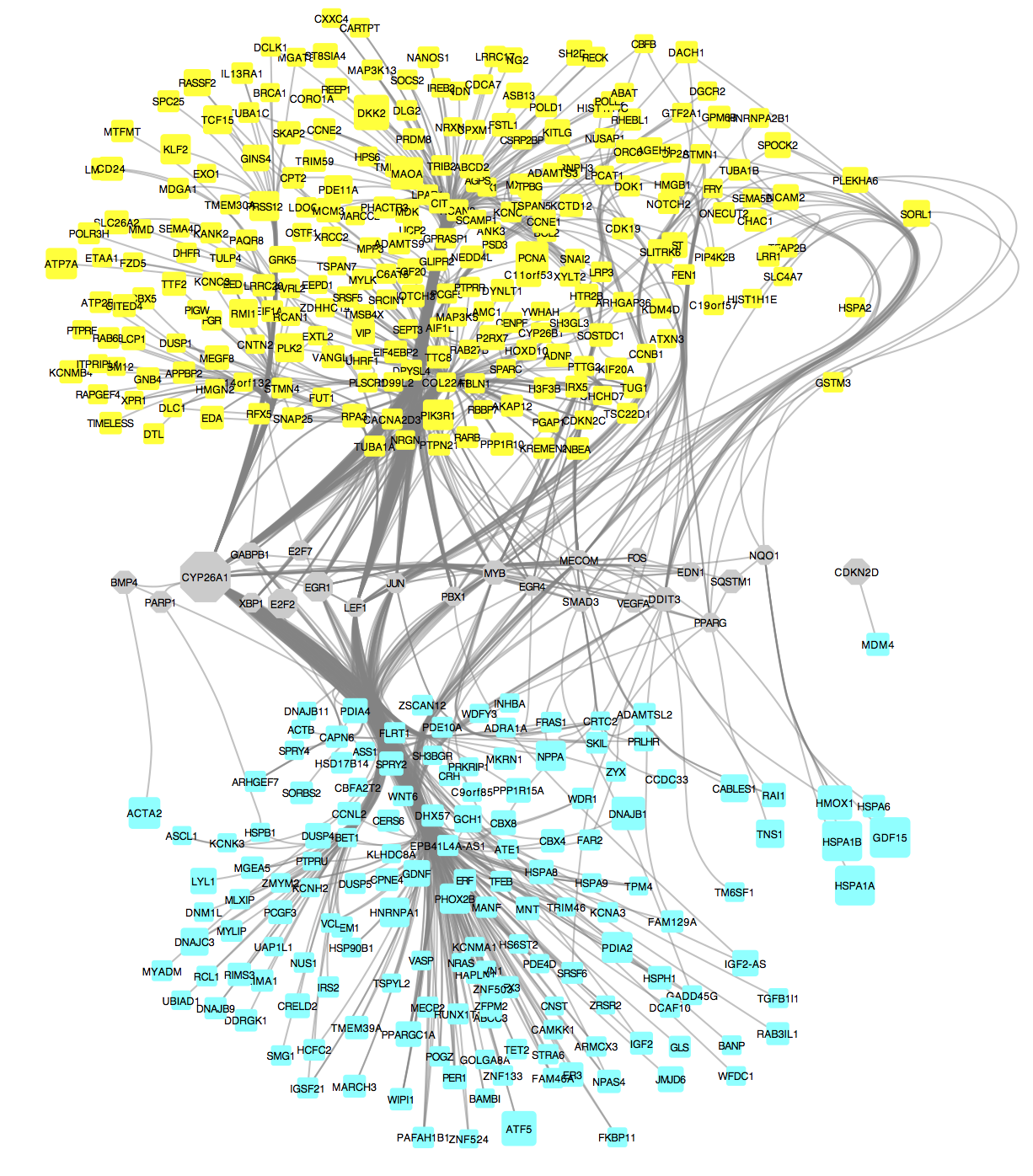


**A**


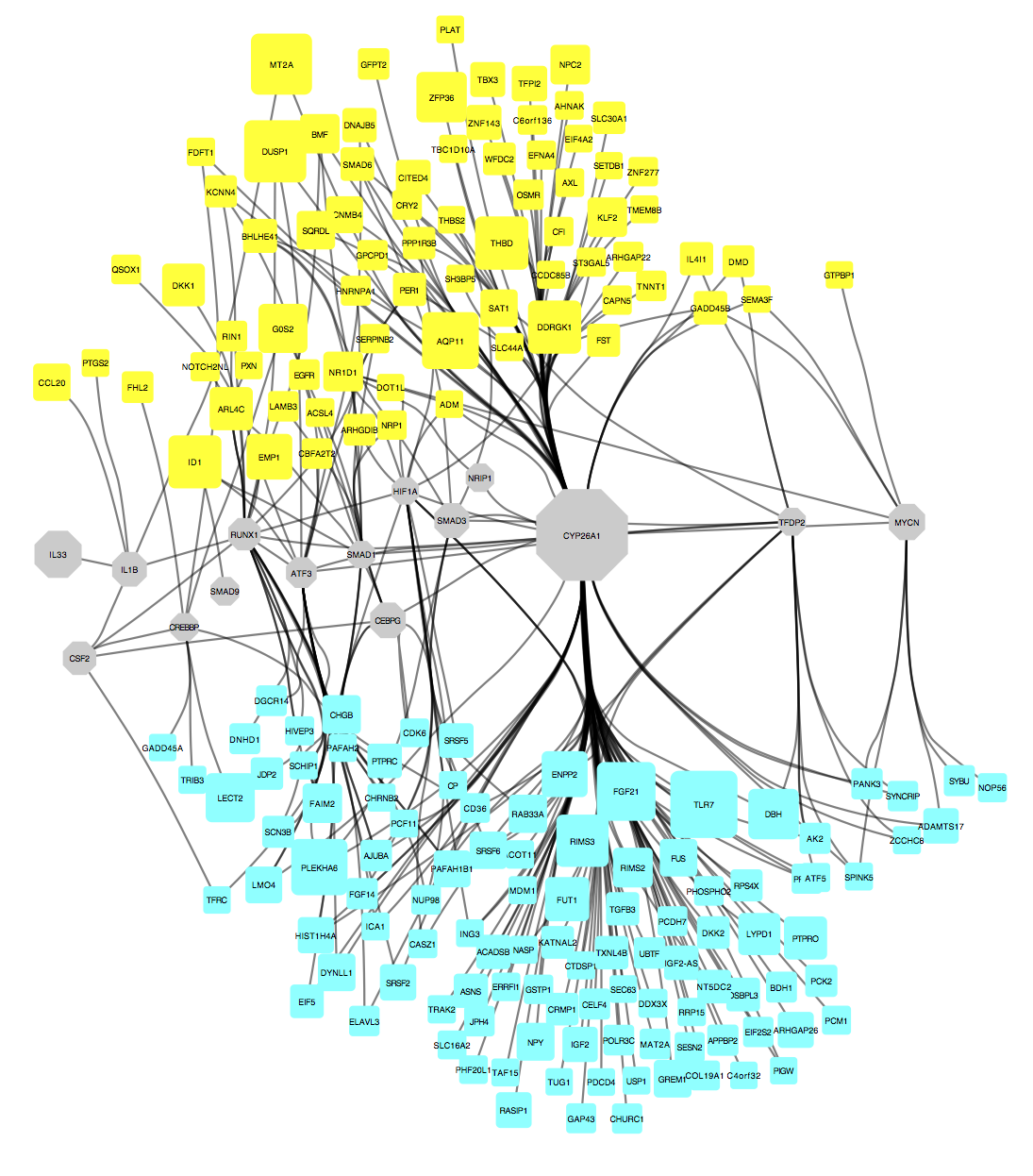
**Figure S6. Gene expression changes can be explained by a small number of regulatory genes.** Network showing up-regulated (yellow) and down-regulated (cyan) genes with previously reported regulatory relationships to genes (grey nodes) in (**A**) SK-N-AS cells (23 genes) and (**B**) SK-N-BE(2)-C cells (15 genes). Nodes were scaled according to absolute expression differences, with larger nodes having more extreme differences in expression in response to treatment.

**B**A


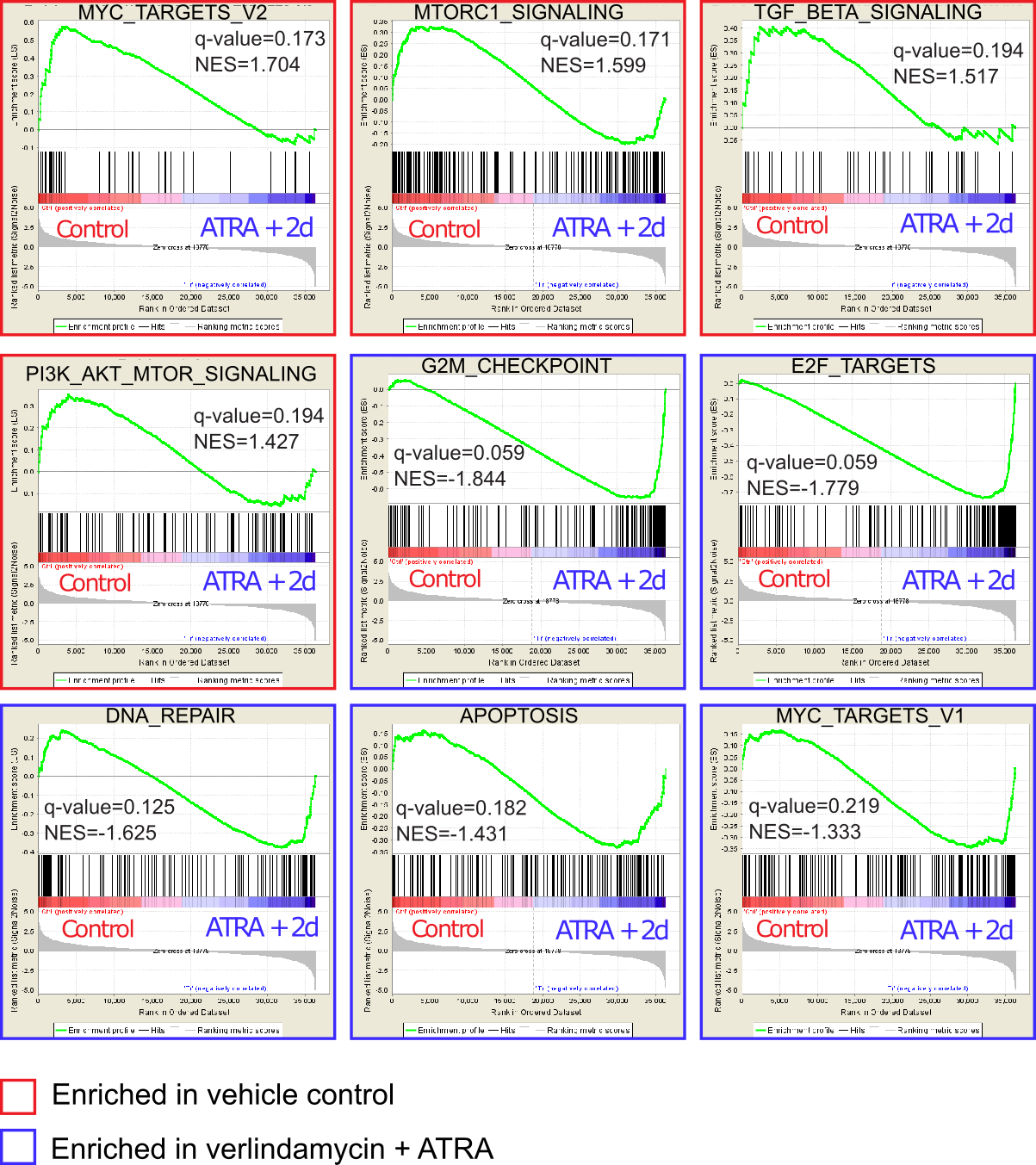


**Figure S7. Gene Set Enrichment Analysis (GSEA) of differential gene expression in SK-N-AS cells following co-treatment with verlindamycin (2d) and ATRA.** Gene expression data generated by expression microarray analysis following 6-days co-treatment with 1 μM ATRA and 0.5 x GI_50_ verlindamycin (2d) versus vehicle (DMSO) control were analyzed using GSEA to extract biological knowledge and highly-significantly enriched gene-sets are shown. The most up-regulated genes in vehicle control are shown on the left side (red), while the most up-regulated genes following ATRA + 2d treatment are shown on the right side (blue). Black bars represent the positions of the vehicle control versus ATRA + 2d up-regulated signature genes in the ranked list. Green curves represent the evolution gene density. Normalized enrichment scores (NES) reflect the degree to which genes are overrepresented. When the distribution is random, the enrichment score is zero. Enrichment of signature genes at the top of the ranked list results in a large positive deviation of the NES from zero. q-value; false discovery rate (FDR)-adjusted q-value.


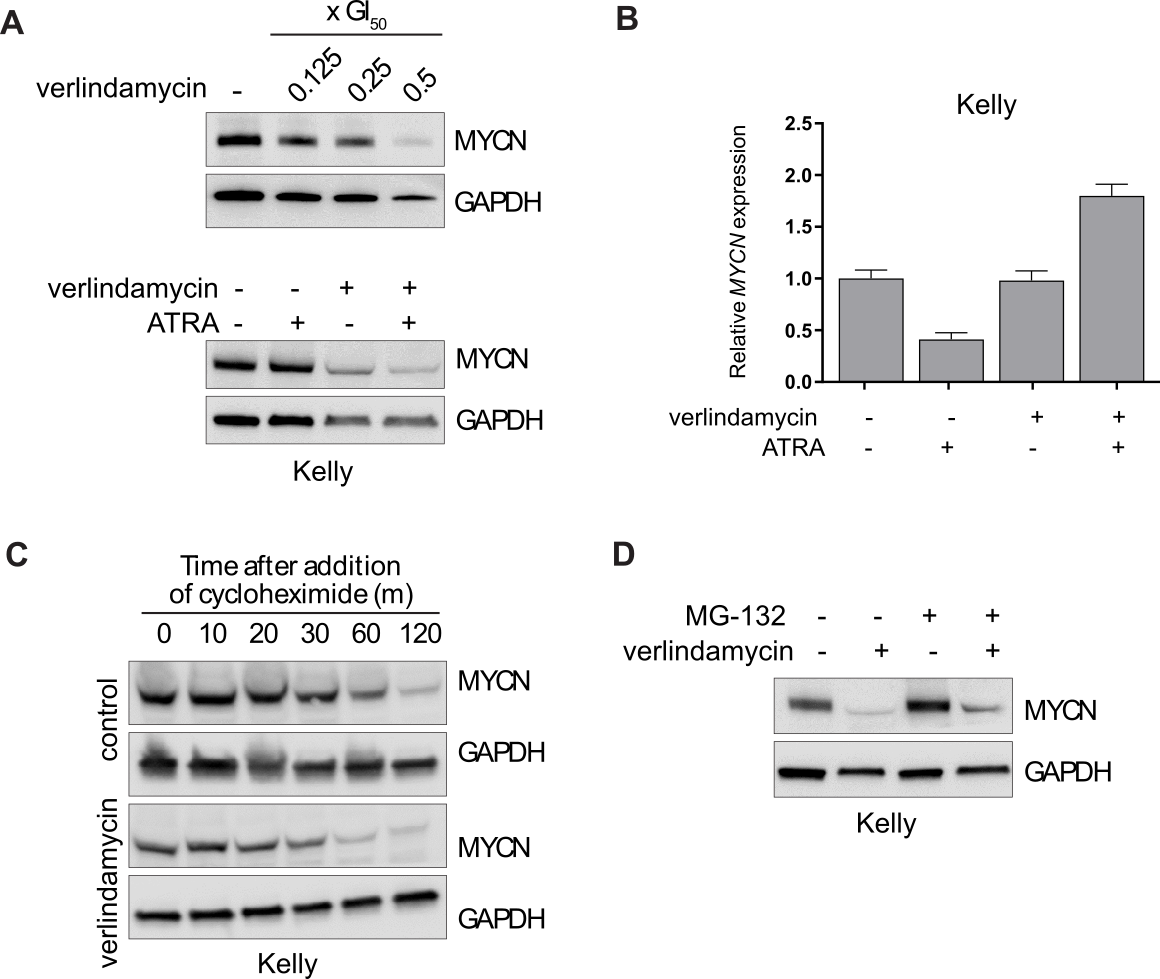


**Figure S8. Verlindamycin down-regulates expression of MYCN protein in *MYCN*-amplified neuroblastoma.** (**A**) MYCN protein levels were assessed in Kelly cells treated for 6 days: with increasing concentrations of verlindamycin (upper panel; GI_50_ = 3.5 μM) or with 0.5 x GI_50_ verlindamycin combined with 1 μM ATRA (lower panel). (**B**) *MYCN* mRNA expression was measured by RT-qPCR (relative to *GAPDH*) in Kelly cells treated for 6 days with 1 μM ATRA, 0.5 x GI_50_ verlindamycin or combination. (**C**) Kelly cells pre-treated with 0.5 x GI_50_ verlindamycin for 4 days were exposed to 25 µg/ml cycloheximide for up to 2h. (**D**) Kelly cells pre-treated with verlindamycin for 4 days were exposed to 10 µM MG-132 for 16h.


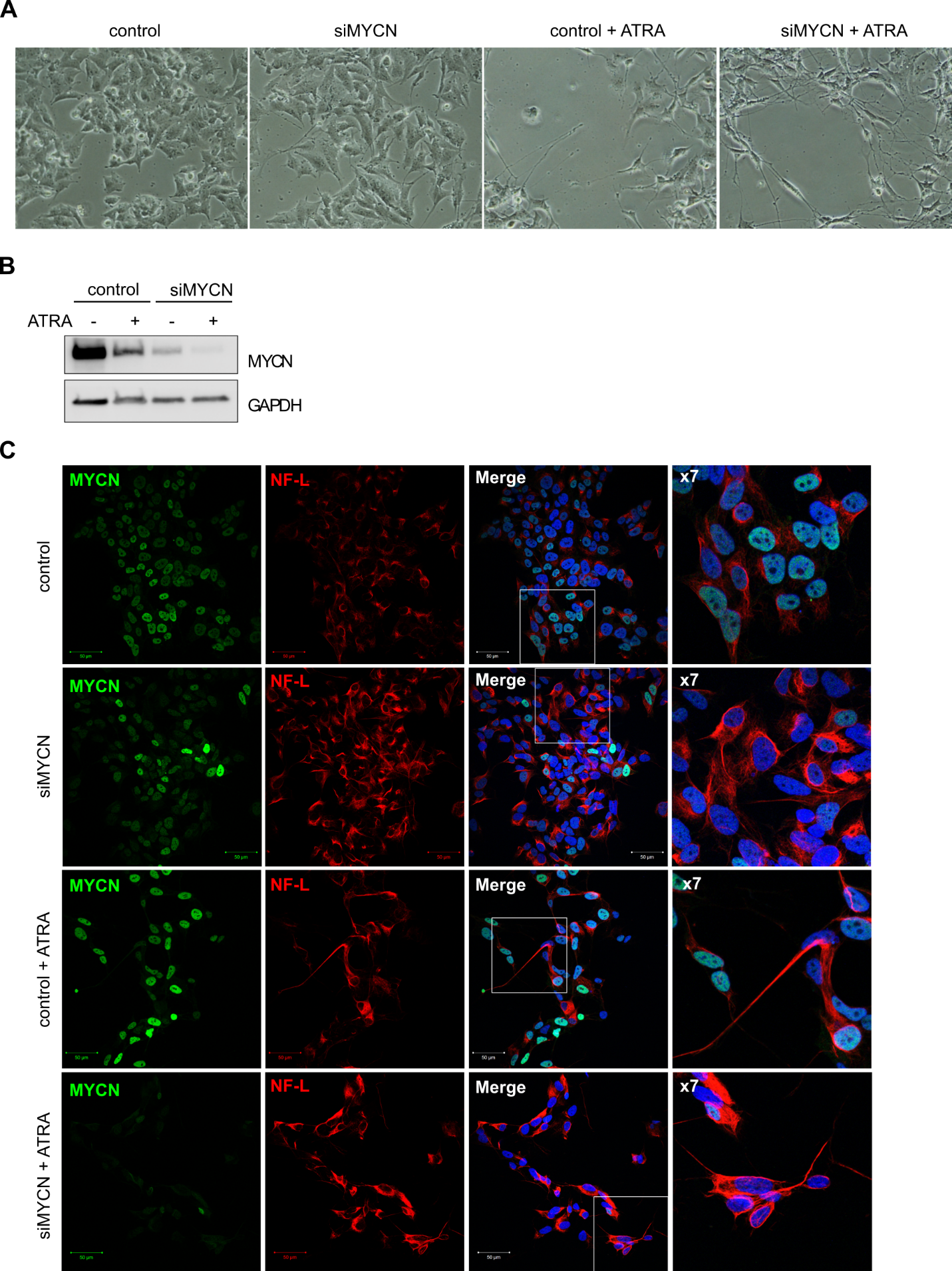


**Figure S9. siRNA-mediated MYCN knock-down leads to neuroblastoma differentiation.** SK-N-BE(2)-C cells were transfected with MYCN-targeting or non-targeting (control) siRNA with or without ATRA treatment for 72h after which (**A**) cells were observed under brightfield microscope (**B**) MYCN protein level was assessed by Western blotting (**C**) cells were fixed and stained with MYCN (green), NF-L (red) and DAPI (blue), scale bar at 50 μm.


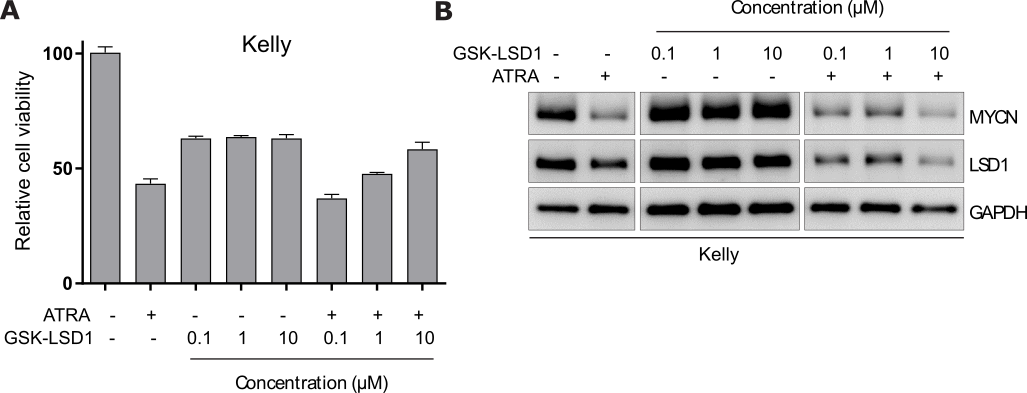


Figure S10. Inhibition of KDM1A/LSD1 is not the mechanism of action of verlindamycin. Kelly cells were treated with increasing concentrations of GSK-LSD1 with or without 1 μM ATRA as indicated for 6 days, after which (A) cell viability was assessed by CellTiter Glo. (B) MYCN expression level was tested in cells treated with GSK-LSD1 with or without addition of ATRA.
